# Accidental Fall Revealing an Atypical Presentation of a Rare Disease: A Case of Budd-Chiari Syndrome

**DOI:** 10.7759/cureus.89061

**Published:** 2025-07-30

**Authors:** Sneha Celine Jossie, Thuraya Al Bhaisi, Farooq Khan

**Affiliations:** 1 Internal Medicine, King's College Hospital London, Dubai, ARE

**Keywords:** budd-chiari syndrome, case report, jak2 mutation, polycythemia vera, protein c deficiency

## Abstract

Budd-Chiari syndrome (BCS) is a rare vascular disorder characterized by hepatic venous outflow obstruction. Polycythemia vera (PV) is a common underlying etiology contributing to BCS. The diagnosis of metabolic dysfunction-associated steatohepatitis (MASH) in this case warrants reconsideration based on recent diagnostic criteria. We report a case of a 53-year-old male patient who presented with weakness, lethargy, and chest pain. A recent fall resulting in a spiral fracture of the thoracic spine led to an incidental diagnosis of BCS, associated with a Janus kinase 2 (JAK2) mutation, protein C deficiency, and PV. Laboratory investigations revealed elevated red blood cell and platelet counts, normal hemoglobin levels, abnormal coagulation parameters, and elevated transaminase levels. The diagnostic workup included a contrast-enhanced abdominal CT scan, an upper gastrointestinal endoscopy, a hepatic vein Doppler ultrasound, and a bilateral lower limb venous Doppler ultrasound. This case underscores the importance of prompt diagnosis and multidisciplinary management of BCS, highlighting PV as a common underlying cause. While MASH was diagnosed based on the clinical and laboratory findings, current hepatology guidelines emphasize histological confirmation as the diagnostic gold standard, warranting further evaluation in such cases.

## Introduction

Budd-Chiari Syndrome (BCS) is a rare, serious condition marked by the obstruction of hepatic venous outflow, leading to dysfunction and potentially life-threatening complications. BCS is most commonly caused by thrombotic occlusion secondary to a chronic myeloproliferative neoplasm [1]. 

The estimated prevalence of BCS ranges from one in 50,000 to one in 100,000 individuals and demonstrates a higher incidence among female patients. A significant proportion of BCS cases are associated with underlying prothrombotic conditions. The most common among these is the Factor V Leiden mutation, present in approximately 53% of patients with BCS [1]. Myeloproliferative neoplasms (MPNs) also constitute a major etiological factor in BCS, particularly in cases involving the Janus kinase 2 (JAK2) V617F somatic mutation. This mutation is identified in 40% to 60% of BCS patients. The World Health Organization classifies MPNs in part based on the presence of this mutation, which plays a critical role in disease pathogenesis [2].

JAK2 encodes a tyrosine kinase involved in cytokine-mediated intracellular signaling. Mutations in this gene are commonly associated with MPNs such as polycythemia vera (PV), essential thrombocythemia, and primary myelofibrosis. PV, in particular, is characterized by clonal erythrocytosis resulting from JAK2-mediated signaling dysregulation, leading to increased blood viscosity and a heightened risk of thromboembolic events, mechanisms that may contribute directly to the development of BCS [3].

## Case presentation

A 53-year-old Jordanian male patient was admitted to the Emergency Department at King’s College Hospital London in Dubai Hills in October 2024. He had a history of a fall in July 2024 that resulted in a spiral fracture of the thoracic spine. A CT scan performed at the time incidentally revealed BCS and a concomitant protein C deficiency. Later that month, he also underwent a cholecystectomy, after which he developed ascites and peripheral edema.

The patient presented to the gastroenterology clinic with complaints of weakness, lethargy, chest pain, and increased abdominal girth. On physical examination, he was oriented, hemodynamically stable, and afebrile. Abdominal distension was noted, with shifting dullness suggestive of ascites. There was tenderness on palpation and pain over the right upper quadrant. Hepatomegaly was palpable, measuring approximately 5-6 cm below the costal margin, along with splenomegaly extending approximately 10 cm. He had no known comorbidities and denied any smoking or alcohol use. There were no known drug allergies.

Serological testing for hepatitis panels returned negative results. Additionally, autoimmune screening, including antiphospholipid, antinuclear, and antineutrophil cytoplasmic antibodies, was negative. Genetic testing for prothrombin gene mutation, Factor V Leiden, and methylenetetrahydrofolate reductase (MTHFR) mutations also showed negative results. These findings helped rule out other potential causes, such as metabolic dysfunction-associated steatotic liver disease (MASLD) and supported the diagnosis of BCS.

He was diagnosed with decompensated liver disease and metabolic dysfunction-associated steatohepatitis (MASH). Prior to the official diagnosis, he was started on Clexane (Enoxaparin) 60 mg, which was then tapered down to 40 mg and finally to 20 mg before diagnostic endoscopy. This tapering was undertaken as a precaution to minimize the risk of gastrointestinal bleeding during the procedure, in line with thromboprophylaxis guidelines that recommend adjusting anticoagulant dosing before invasive interventions. 

Laboratory data indicated elevated RBCs, normal hemoglobin, no leukocytosis, and abnormal coagulation panels with elevated transaminases and partial thromboplastin time (PTT) counts (Table 1).

**Table 1:** Laboratory data RBC: red blood cell; WBC: white blood cell; MCV: mean corpuscular volume; MCH: mean corpuscular hemoglobin; MCHC: mean corpuscular hemoglobin concentration; RDW: red blood cell distribution width; Hgb: hemoglobin; AST: aspartate aminotransferase; ALT: alanine aminotransferase; CRP: C-reactive protein; INR: international normalized ratio; PT: prothrombin time; aPTT: activated partial thromboplastin time; ↑: greater than normal values; ↓: Lesser than normal values; — : similar to normal values.

Test	Result	Normal range
RBC (x10^6^/uL)	7 ↑	4.14-5.8
MCV (fL)	71 ↓	79-97
MCH (pg)	22 ↓	27-33
Hgb (g/dL)	15.4 —	12.6-17.7
Hematocrit (%)	49.5 —	37.5-51.0
MCHC (g/dL)	31 ↓	32-36
RDW (%)	20.9 ↑	12.3-15.4
WBC (×10^3^/µL)	5.5 —	3.4-10.8
Platelets (×10^3^/µL)	385 ↑	150-379
Sodium (mmol/L)	137 —	134-144
Potassium (mmol/L)	4.7 —	3.5-5.2
Creatinine (mg/dL)	0.89 —	0.76-1.27
Glucose (mg/dL)	87 —	50-450
Hgb A1C	5.9 ↑	4.8-5.6
Total proteins (g/dL)	8.7 ↑	6.0-8.5
Albumin (g/dL)	4.4 —	3.5-5.5
AST (U/L)	29 —	0-40
ALT (U/L)	24 —	0-44
Total bilirubin (mg/dL)	1.73 ↑	0-1.2
Globulin (g/dL)	4.3 ↑	2.3-3.5
Direct bilirubin (mg/dL)	0.68 ↑	0-0.4
Alkaline phosphatase (U/L)	206 ↑	39-117
Protein C	52 ↓	73-180
Protein S	66 —	63-140
Antithrombin III	80 —	75-135
PT (seconds)	37.7 ↑	11.7-15.3
INR	3.11 ↑	0.8-1.2
aPTT (seconds)	50.9 ↑	26-40
CRP (mg/L)	3.4 —	0-4.9

**Figure 1:**
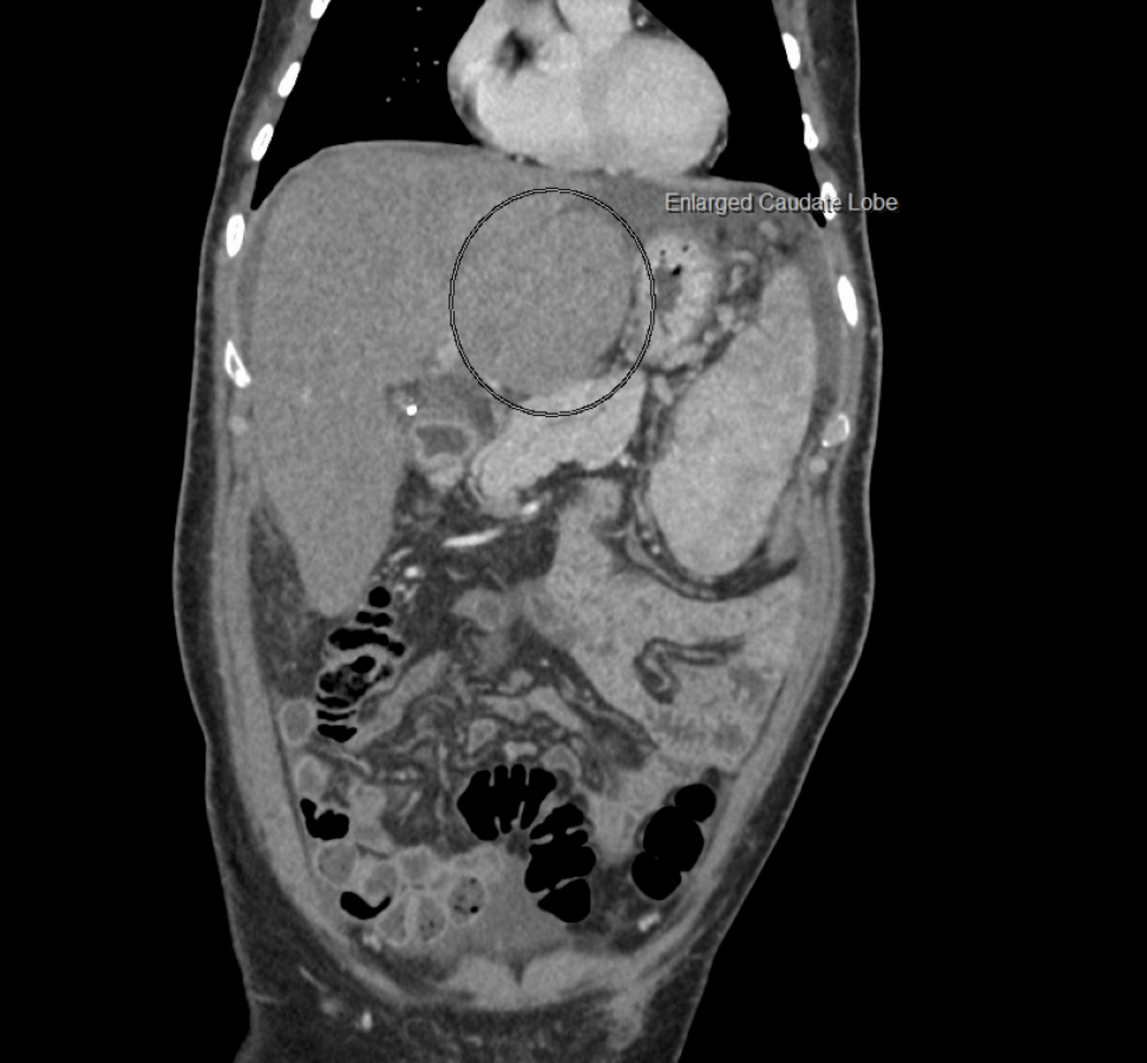
Abdominal CT image showed an enlarged caudate lobe (as indicated within the circle)

**Figure 2:**
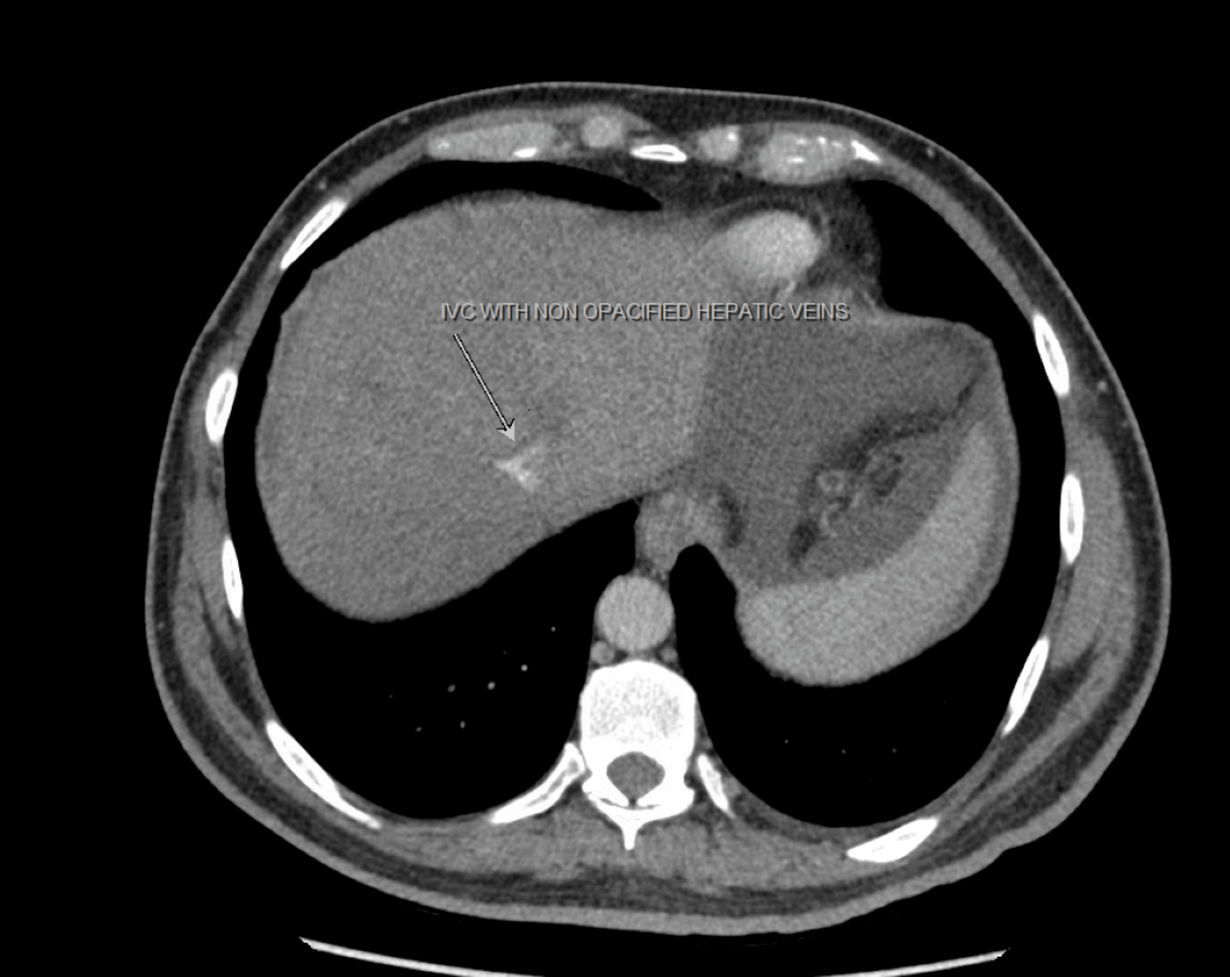
Abdominal CT with contrast showed the attenuation of hepatic veins (marked by the white arrow)

**Figure 3:**
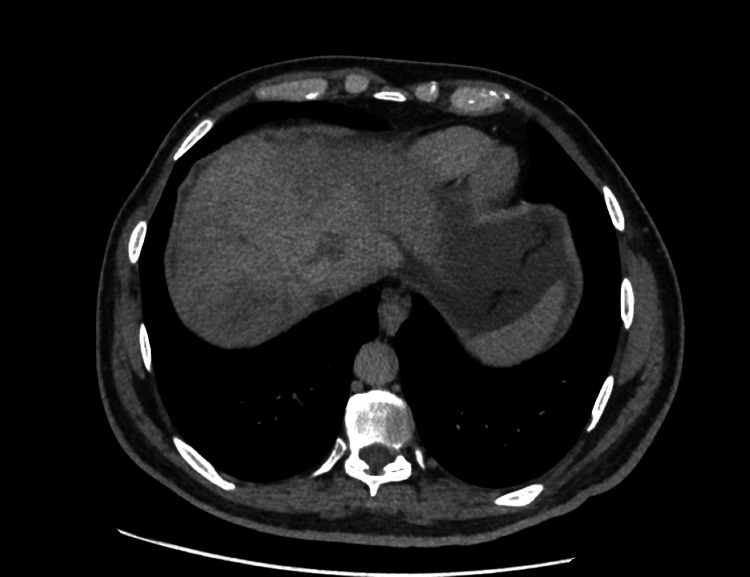
CT abdomen showed the general mild appearance of nutmeg liver that is characteristic of Budd Chiari syndrome (BCS)

A CT scan of the abdomen revealed hepatomegaly with hypertrophy of right caudate lobe (Figure 1), non-opacified hepatic veins, and heterogeneous parenchyma at the hepatic dome suggestive of BCS (Figures 2, 3).

**Figure 4:**
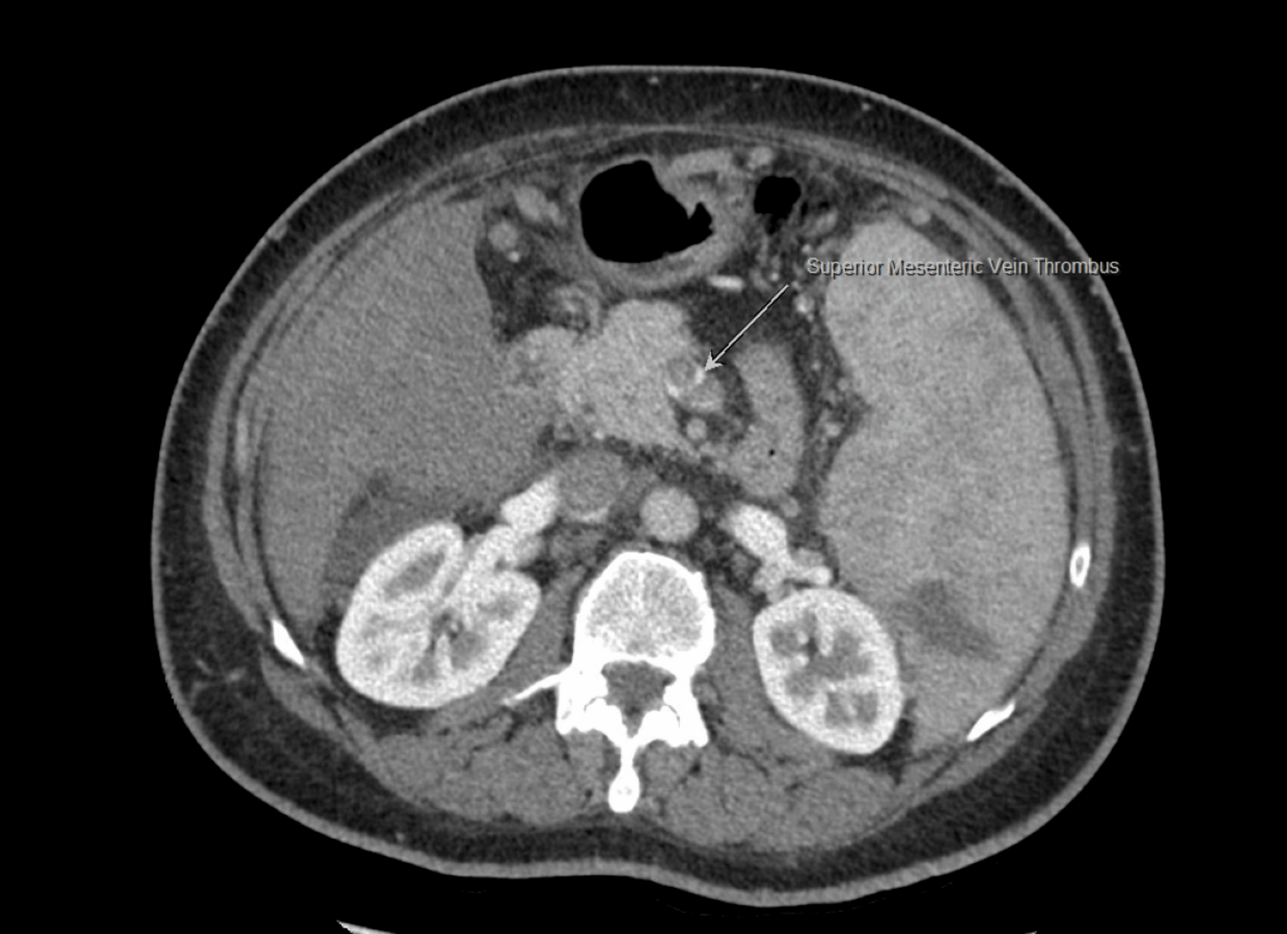
CT abdomen revealed a thrombus in the superior mesenteric vein (marked by the white arrow)

There was also a filling defect observed, involving the superior mesenteric vein (SMV), and the main and left portal veins, along with partial thrombosis (Figure 4).

**Figure 5:**
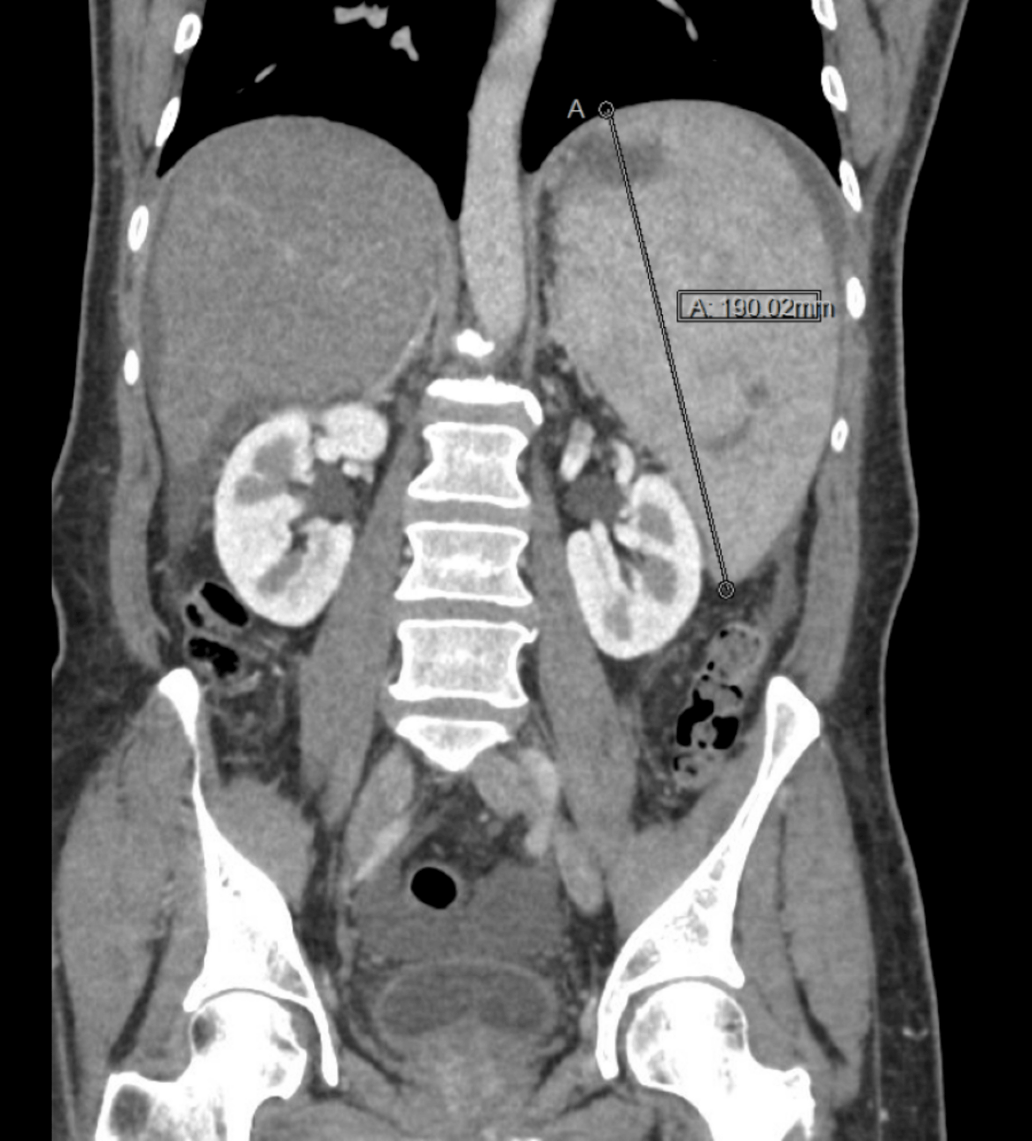
CT abdomen revealed an enlarged spleen of about 190 mm (measurement indicated by the black line)

**Figure 6:**
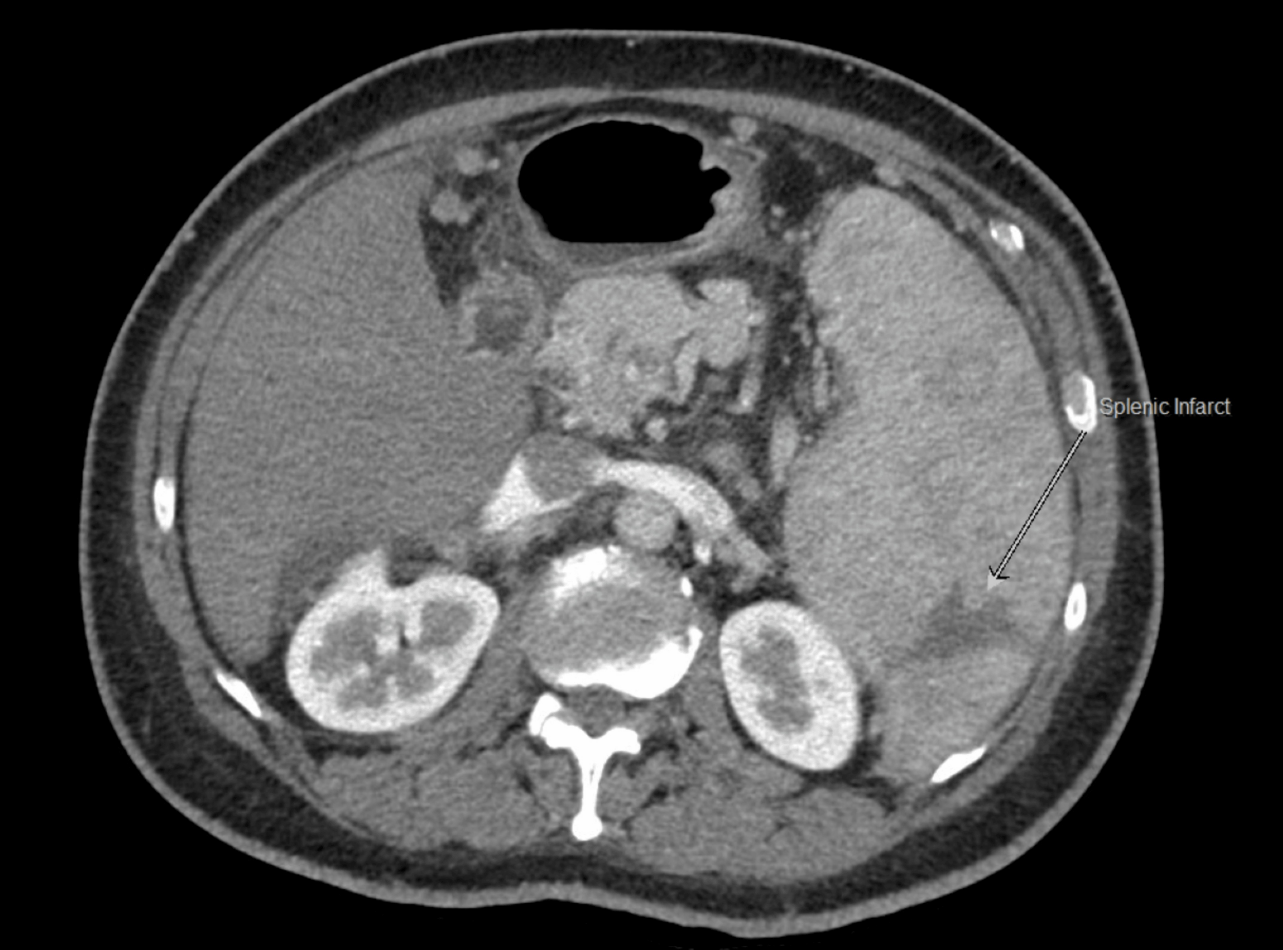
Abdominal CT image revealed a wedge-shaped splenic infarct (as indicated by the grey arrow)

A markedly enlarged spleen with a lower polar subcapsular branching non-enhancing lesion, likely suggestive of a splenic infarct, was also observed (Figures 5, 6).

**Figure 7:**
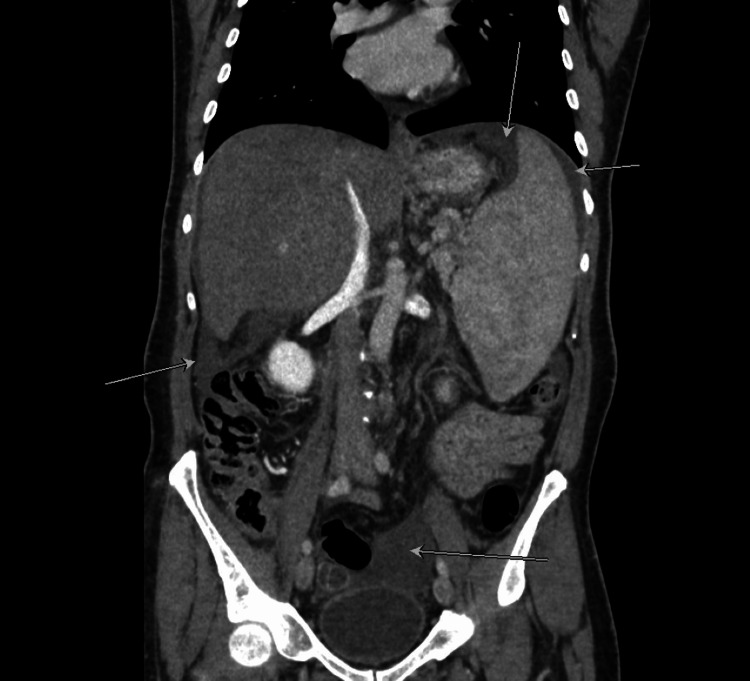
Contrast-enhanced abdominal CT revealed the accumulation of free fluid (ascites), highlighted by the white arrows

The contrast-enhanced abdominal CT revealed mild-to-moderate free ascites (Figure 7).

**Figure 8:**
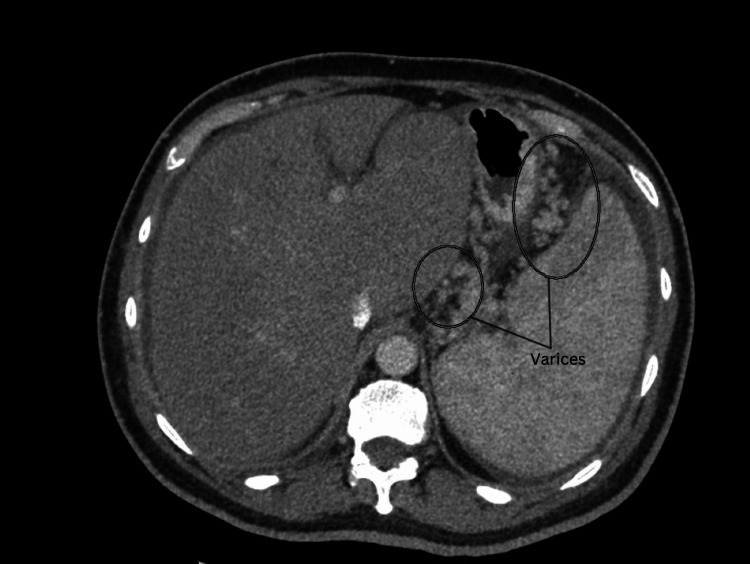
Abdominal CT image clearly showed the presence of varices (as indicated by the circles)

The contrast-enhanced abdominal CT demonstrated prominent enhancing collateral vessels in the region of the gastroesophageal junction, consistent with varices (Figure 8).

**Figure 9:**
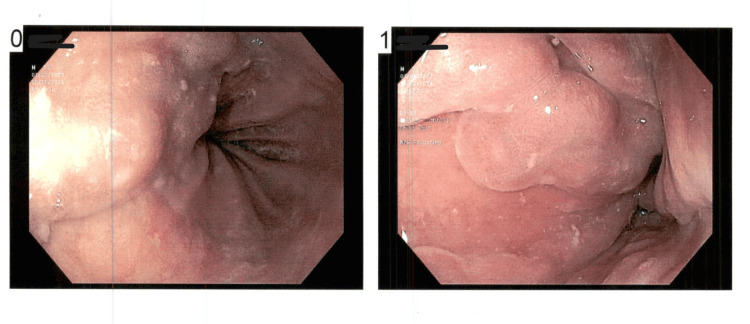
Endoscopy images (denoted by 0 and 1) revealed grades two and three varices, respectively

Upper gastrointestinal endoscopy revealed two columns of grade two and one of grade three esophageal varices (Figure 9).

Two bands were applied with good proximal decompression.

**Figure 10:**
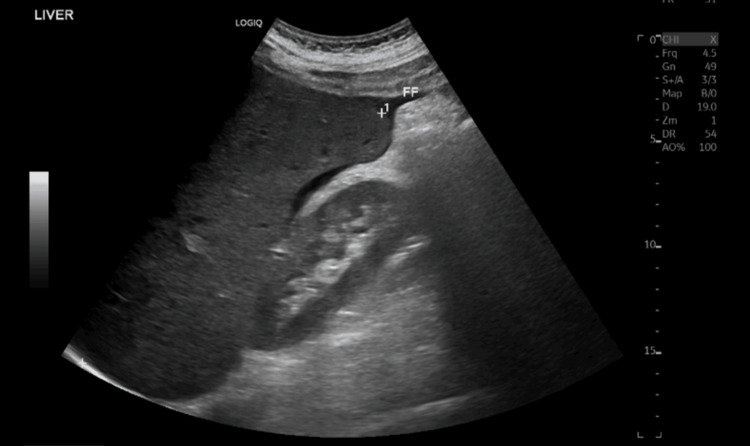
An ultrasound hepatic vein doppler showed hepatomegaly of about 18 cm

**Figure 11:**
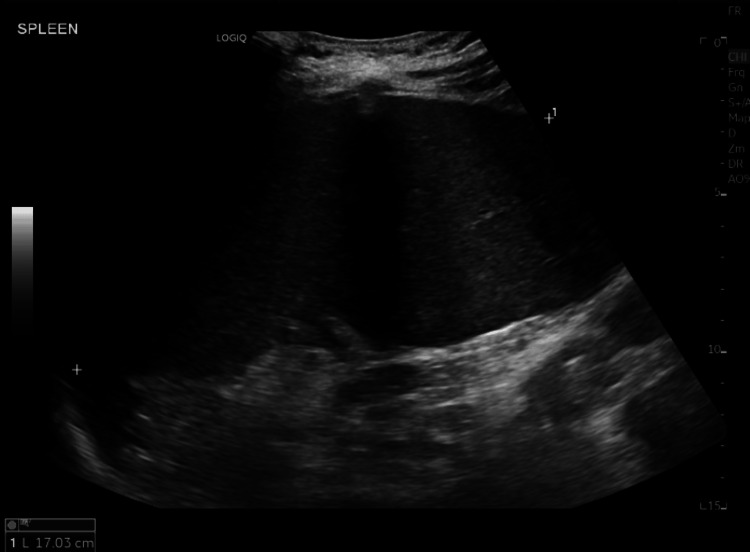
An ultrasound hepatic vein doppler showed splenomegaly of about 17 cm

**Figure 12:**
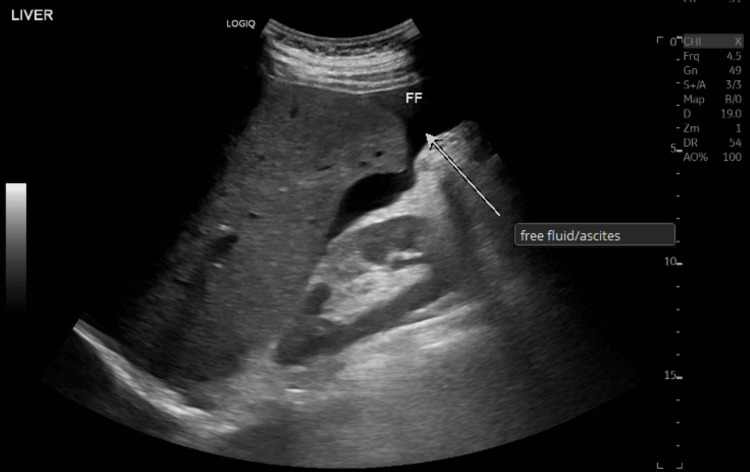
An ultrasound hepatic vein doppler revealed an accumulation of free fluid (indicated by the white arrow)

A hepatic vein doppler ultrasound revealed hepatosplenomegaly (Figures 10, 11) with mild pelvi-abdominal ascites (Figure 12).

**Figure 13:**
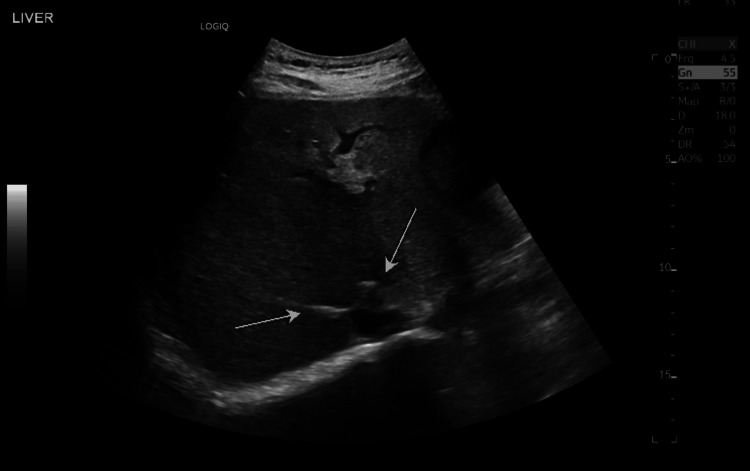
An ultrasound hepatic vein doppler revealed attenuated hepatic veins (indicated by the white arrows)

**Figure 14:**
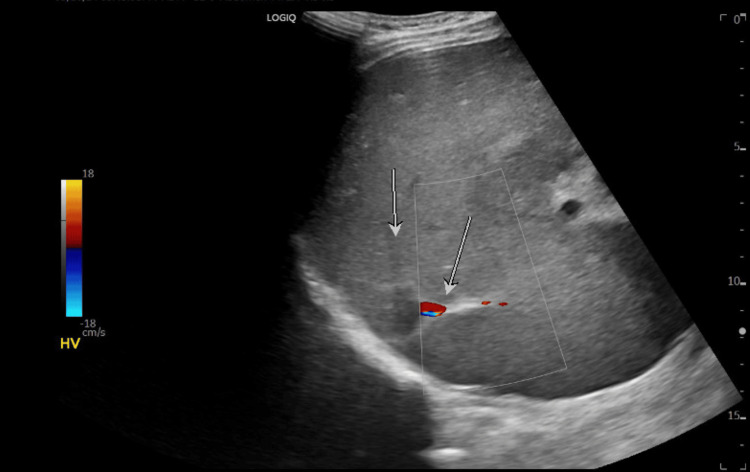
Attenuated hepatic veins (indicated by the white arrows; flow seen with the color)

A relative attenuation of hepatic veins was also seen (Figures 13, 14).

A bilateral lower limb venous doppler revealed patent deep venous system with no evidence of filling defects/luminal obstruction. The preserved venous compressibility indicated no evidence of deep venous thrombosis (DVT).

Post-procedure, the patient was diagnosed with primary BCS. Enoxaparin was discontinued and replaced with oral rivaroxaban 15 mg, and the patient was prescribed carvedilol 6.25 mg, spironolactone 25 mg, and furosemide 40 mg. Carvedilol was initiated to reduce portal pressure, as the Doppler ultrasonography revealed a patent portal vein measuring 11 mm in diameter with a peak systolic velocity (PSV) of 15 cm/s, slightly below the normal range of 16-40 cm/s, suggesting early portal hypertension. However, no follow-up Doppler measurements or invasive portal pressure assessments were available after treatment initiation. Hydroxyurea 500 mg and allopurinol 300 mg were started to target suspected PV, and nefopam 30 mg was administered for abdominal pain management.

A dietitian was consulted for dietary modifications, and the patient was advised a low salt diet with carbohydrate snacks of approximately 50 gm at night, and was advised iron, thiamine, and B complex supplements.

The JAK2 mutation analysis subsequently returned positive, confirming the diagnosis of PV. The patient remained stable during hospitalization and was discharged with scheduled follow-ups in gastroenterology, hematology, and oncology. Invasive procedures were not indicated at the time. Long-term anticoagulation with rivaroxaban was continued at discharge, and he remains a viable candidate for liver transplantation.

## Discussion

The case highlights the complex interplay between BCS and PV in a rare presentation in a male patient. We also emphasize the significance of a multidisciplinary approach to diagnosis and treatment.

The etiology of BCS remains unclear in most cases. In our patient, primary idiopathic BCS was diagnosed, characterized by thrombosis and hepatic venous outflow obstruction, leading to liver congestion and subsequent ischemic necrosis [4].

The severity of BCS largely depends on the extent and rapidity of venous occlusion. In the setting of acute thrombosis or hepatic dysfunction, levels of natural anticoagulants, such as protein C, protein S, and antithrombin III, may be reduced. Since these proteins are synthesized by the liver, hepatic synthetic impairment can complicate the differentiation between inherited and acquired deficiencies. Current recommendations suggest that testing for hereditary thrombophilias be delayed until hepatic function stabilizes and the acute thrombotic episode has resolved, ideally six to 12 weeks after discontinuing anticoagulation. Repeat testing and familial screening may be required to confirm inherited thrombophilias. Among these, protein C deficiency is most frequently implicated in BCS, with a reported prevalence of up to 25% [1,2,5].

Imaging is of utmost importance for the timely diagnosis of BCS. Typical findings such as a nutmeg appearance of the liver with an enlarged caudate lobe, the presence of thrombi, and attenuation of the hepatic veins are highly suggestive and were observed in our patient [6]. 

In this case, the patient had abdominal pain, ascites, and increased abdominal girth. After investigations and multiple lab tests, findings were suggestive of PV alongside the initial diagnosis of BCS.

Management of BCS should follow a stepwise algorithm. First-line treatment includes lifelong anticoagulation, management of the underlying cause, and control of portal hypertension. If inadequate, second-line options include angioplasty or stenting, followed by Transjugular Intrahepatic Portosystemic Shunt (TIPS) in persistent or recurrent cases. Liver transplantation remains the final recourse in patients with hepatic decompensation or treatment failure [5]. Transplant eligibility and timing are guided by prognostic scoring systems such as the Model for End-Stage Liver Disease (MELD) and the Child-Pugh classification, which assist in assessing the severity of liver dysfunction and prioritizing referral [7].

At the time of discharge, the patient was clinically stable. However, due to the chronic nature of both PV and BCS, long-term monitoring and regular follow-up are essential to manage potential relapses. A similar case reported by Kulkarni et al. [8] described a 49-year-old male patient with BCS secondary to PV who underwent percutaneous recanalization with favorable long-term outcomes. This highlights the variable clinical course of BCS and the importance of individualized management strategies.

## Conclusions

The purpose of this case study is to highlight the occurrence of a rare condition, such as BCS, in a male patient, as opposed to its common occurrence in females, and to exemplify the importance of how extensive laboratory and serological tests are required to reach its diagnosis. It is also important to identify any other underlying etiologies, PV in this case, as well as the consideration of other inherited or acquired thrombophilias. A thorough differential workup should include screening for JAK2 mutations, protein C/S, and antithrombin III levels, and where indicated, family history assessment and genetic testing to distinguish inherited deficiencies. Timely and multidisciplinary management is required to restore hepatic venous flow, with consistent follow-up and the possibility of liver transplantation in deteriorating cases.
